# Metronidazole exposure–response and safety in infants

**DOI:** 10.1128/aac.00377-25

**Published:** 2025-09-19

**Authors:** Rachel L. Randell, Stephen J. Balevic, Rachel G. Greenberg, Michael Cohen-Wolkowiez, Michael J. Smith, Daniel K. Benjamin Jr., Catherine Bendel, Joseph M. Bliss, Hala Chaaban, Rakesh Chhabra, Christiane E. L. Dammann, L. Corbin Downey, Chi D. Hornik, Naveed Hussain, Matthew M. Laughon, Adrian Lavery, Fernando Moya, Matthew Saxonhouse, Gregory M. Sokol, Andrea Trembath, Joern-Hendrik Weitkamp, Christoph P. Hornik, on behalf of the Best Pharmaceuticals for Children Act—Pediatric Trials Network Steering Committee, Daniel K. Benjamin,

**Affiliations:** 1 Duke Clinical Research Institute, Durham, North Carolina, USA; 2 Duke University Medical Center, Durham, North Carolina, USA; 3 Independent Researcher, Scottsdale, AZ, USA; 4 University of North Carolina at Chapel Hill, Chapel Hill, North Carolina, USA; 5 Penn State College of Medicine, Hershey, Pennsylvania, USA; 6 University of Louisville, Louisville, Kentucky, USA; 7 Children's Hospital of Philadelphia, Philadelphia, Pennsylvania, USA; 8 Wichita Medical Research and Education Foundation, Wichita, Kansas, USA; 9 International Children’s Advocacy Network; The Eunice Kennedy Shriver National Institute of Child Health and Human Development (NICHD), Bethesda, Maryland, USA; 10 The Emmes Company, LLC (Data Coordinating Center), Rockville, Maryland, USA; 1 Department of Pediatrics, Duke University 3065, Durham, North Carolina, USA; 2 Duke Clinical Research Institute 169142, Durham, North Carolina, USA; 3 Department of Pediatrics, University of Minnesota Medical School 12269, Minneapolis, Minnesota, USA; 4 Department of Pediatrics, Division of Neonatology, University of Rochester Medical Center 6923, Rochester, New York, USA; 5 Department of Pediatrics, Division of Neonatology, Oklahoma University Health Sciences Center 173793, Oklahoma City, Oklahoma, USA; 6 Department of Pediatrics, Division of Neonatology, Hackensack University Medical Center, Hackensack Meridian School of Medicine 3673, Hackensack, New Jersey, USA; 7 Department of Pediatrics, Tufts Medical Center, Tufts University, Boston, Massachusetts, USA; 8 Department of Pediatrics, Atrium Health Wake Forest Baptist Medical Center 12280, Winston-Salem, North Carolina, USA; 9 Department of Pediatrics, Division of Neonatology, Connecticut Children’s 129552, Hartford, Connecticut, USA; 10 Department of Pediatrics, Division of Neonatal-Perinatal Medicine, The University of North Carolina at Chapel Hill 2331, Chapel Hill, North Carolina, USA; 11 Children’s Hospital of Orange County, Orange, California, USA; 12 Pediatrix Medical Group, Austin, Texas, USA; 13 Department of Pediatrics, Division of Neonatology, Levine Children’s Hospital, Wake Forest School of Medicine Charlotte Campus, Atrium Healthcare, Charlotte, North Carolina, USA; 14 Department of Pediatrics, Indiana University School of Medicine 12250, Indianapolis, Indiana, USA; 15 Division of Neonatal-Perinatal Medicine, The University of North Carolina at Chapel Hill 2331, Chapel Hill, North Carolina, USA; 16 Mildred Stahlman Division of Neonatology, Monroe Carell Jr. Children’s Hospital at Vanderbilt, Vanderbilt University Medical Center, Nashville, Tennessee, USA; University Children's Hospital, Münster, Germany

**Keywords:** metronidazole, pediatric, pharmacodynamics, safety

## Abstract

The nitroimidazole antibiotic, metronidazole, is frequently prescribed to infants with serious intra-abdominal infections, and multiple dosing recommendations exist. We sought to evaluate the extent to which metronidazole doses and associated exposures achieved desired efficacy and safety in infants enrolled in the Antibiotic Safety in Infants with Complicated Intra-abdominal Infections (SCAMP) trial (NCT01994993). SCAMP participants received intravenous metronidazole as part of multimodal antimicrobial therapy. Participants received a 15 mg/kg loading dose and a 7.5 mg/kg maintenance dose at 24 h. A subsequent 7.5 mg/kg maintenance dose was administered every 12 h for participants of postmenstrual age (PMA) 23 to <34 weeks; 8 h for PMA 34–40 weeks; and 6 h for PMA >40 weeks. We evaluated associations between simulated metronidazole exposures and pre-specified surrogate pharmacodynamic targets and clinical outcomes of efficacy and safety. Nearly 100% of pharmacodynamic targets were met. Infants with therapeutic success (a composite efficacy outcome, defined as the absence of death, negative bacterial blood cultures, and presumptive clinical cure at 30 days) had higher *C*_min,ss_, *C*_max,ss_, AUC0_0–24,ss_, and AUC_cum_ compared with infants without therapeutic success. However, the relationships between these exposure measures and therapeutic success were not significant in logistic regression analysis adjusting for gestational age. Despite generally high simulated exposures, no relationships were observed between exposures and prespecified safety events (necrotizing enterocolitis, intestinal strictures, intestinal perforation, positive blood culture, seizures, death, and intraventricular hemorrhage). Findings support metronidazole dosing as administered in term and preterm infants in the SCAMP trial.

## INTRODUCTION

The nitroimidazole antibiotic, metronidazole, is frequently used to treat infections in infants (1–4) but dosing is complicated because pharmacokinetic (PK) parameters (5–7) and metabolism (7,8) vary by postnatal age (PNA), gestational age (GA), or postmenstrual age (PMA). Several dosing recommendations exist (9); only the recommendations by Cohen-Wolkowiez et al. (2,10) and Suyagh et al. (6) include PK modeling and exposure–response analysis. However, both exposure–response analyses relied upon one or two surrogate efficacy target(s), which varied across studies and lacked clinical efficacy data. Both investigator groups recommend a 15 mg/kg loading dose followed by differing weight-based maintenance doses ranging from 7.5 to 10 mg/kg with PMA-based intervals ranging from every 6–24 h. To date, only one study has examined the association between metronidazole exposure and safety in infants, and findings were limited to a single, rare safety event (seizures) (11). Thus, there is a need to understand whether published metronidazole dosing recommendations are likely to achieve desired efficacy and safety in infants.

We recently validated the Cohen-Wolkowiez et al. pediatric population pharmacokinetic (popPK) model of metronidazole (10) using opportunistic dried blood spots (12) collected in the multicenter Antibiotic Safety in Infants with Complicated Intra-abdominal Infections (SCAMP) trial (NCT01994993) (13). We also optimized the model to characterize popPK of metronidazole across a wider range of infant ages than previously reported. The optimized model contains a sigmoidal *E*_max_ maturation function of PMA on clearance and estimated exponent of weight on volume of distribution and adequately characterized metronidazole popPK for preterm and term infants ranging from 22.7 to 41.0 weeks GA and 23 to 48 weeks PMA (12).

The objective of this study was to investigate metronidazole exposure–response and safety using the optimized popPK model. We compared simulated metronidazole exposures with multiple pre-specified surrogate pharmacodynamic targets, clinical efficacy outcomes, and safety events collected in SCAMP to evaluate exposure–response and exposure–safety of metronidazole in infants.

## MATERIALS AND METHODS

### Data source

We included 122 SCAMP trial participants who received metronidazole and contributed at least one PK sample. SCAMP trial methods are previously reported (13), and demographics of the 122 participants included in this analysis are detailed in our prior publication; see Table S1 in reference 12. Briefly, most participants were critically ill preterm neonates (mean [SD] GA 31.4 [5.1] weeks, PNA 16.7 [15.8] days, and PMA 33.8 [5.4] weeks) with mean (SD) weight 1.9 (1.0) kilograms who had suspected or confirmed intra-abdominal infections; however, some term and older infants with a range of infections also enrolled in the trial. SCAMP participants received intravenous metronidazole dosing per the Cohen-Wolkowiez et al. recommendations (10) as part of multimodal antimicrobial therapy. The metronidazole loading dose was a 15 mg/kg, 30-min infusion, followed by the first 7.5 mg/kg maintenance dose 24 h after the start of the loading dose. Subsequent maintenance doses were administered as 7.5 mg/kg every 12 h for PMA 23 to <34 weeks, every 8 h for PMA 34–40 weeks, and every 6 h for PMA >40 weeks.

### Exposure simulations

We simulated exposures because metronidazole shows high pharmacokinetic variability in infants (6), and samples were obtained opportunistically in SCAMP (12). Individual participant exposures were simulated using the actual metronidazole dosing in SCAMP and empirical Bayesian estimates of PK parameters from an optimized metronidazole pediatric popPK model, which included participant demographics as covariates (12). Simulated exposure parameters were maximum concentration after the loading dose (C_max,1_), minimum concentration after the loading dose (C_min,1_), area under the concentration–time curve from 0 to 24 h after the loading dose (AUC_0–24,1_), maximum concentration at steady state (C_max,ss_), minimum concentration at steady state (C_min,ss_), and steady-state area under the concentration–time curve from 0 to 24 h (AUC_0–24,ss_).

Equations for calculating C_max,1_, C_min,1_, and AUC_0–24,1_ are as follows:


(1) $$\begin{array}{c} C_{max,1}=\;\frac{Dose}{\left(CL*DUR\right)}*\left(1-e^{-K_{e\;}*\;DUR}\right) \end{array}$$

(2) $$\begin{array}{c} C_{min,1}=\;C_{max,1}*\;e^{-K_{e}\;*\;\left(24-DUR\right)} \end{array}$$

(3) $$\begin{array}{c} {AUC}_{0-24,1}=\frac{Dose}{CL}-\frac{C_{min,1}}{K_{e}} \end{array}$$

where K_e_ denotes the first-order elimination rate constant calculated as CL/*V* (where CL = clearance and *V* = volume) and DUR denotes the infusion duration (0.5 h for all dosing simulations).

Equations for calculating C_max,ss_, C_min,ss_, and AUC_0–24,ss_ are as follows:


(4) $$\begin{array}{c} C_{max,ss}=\;\frac{Dose}{\left(CL*DUR\right)}*\frac{\left(1-e^{-K_{e}\;*\;DUR}\right)}{\left(1-e^{-K_{e}\;*\;\tau }\right)} \end{array}$$

(5) $$\begin{array}{c} C_{min,ss}=C_{max,ss}*e^{-K_{e}\;*\;\left(\tau \;-\;DUR\right)} \end{array}$$

(6) $$\begin{array}{c} {AUC}_{0-24,ss}=\frac{Dose}{CL}*\frac{24}{\tau } \end{array}$$

where *K*_e_ denotes the first-order elimination rate constant calculated as CL/*V;* DUR denotes the infusion duration (0.5 h for all dosing simulations); and *τ* is the dosing interval.

For each SCAMP participant, AUC_0–24,ss_, C_max,ss_, and C_min,ss_ were calculated for individual study doses, beginning 24 h after the first (loading) dose through the end of the study period. Exposure calculations were then averaged to define the individual participants’ exposure over the entire study period.

Exposure corresponding to each efficacy and safety event was calculated as AUC from the time of the first dose to the time of event in days (AUC_cum_). The equation for this is as follows:


(7) $$\begin{array}{c} {AUC}_{cum}=\int_{0}^{t_{e}}C_{p}dt \end{array}$$

where *C*_*p*_ is the simulated plasma concentration (12); *t*_e_ is the time of event in days after the first dose, and *t* is the time after the first dose. For participants who did not have these events, *t*_e_ represents the time of the entire study period (up to 100 days).

### Surrogate pharmacodynamics

We examined the following surrogate pharmacodynamic targets: C_min,ss_ > 8 mg/L, C_min,ss_ >2 mg/L, and the ratio of AUC_0–24,ss_ to the minimum inhibitory concentration (MIC) ≥ 70 for MIC 2 µg/mL and MIC 4 µg/mL. We examined multiple targets because the optimal pharmacodynamic target for metronidazole in infants with intra-abdominal infections is not defined. C_min,ss_ was selected as a conservative measure of exposure that has been used in prior dose-optimization studies of metronidazole in infants (2, 6, 10). The Clinical and Laboratory Standards Institute (14) defines 8 mg/L or less as the breakpoint of metronidazole for *Bacteroides fragilis*, an anaerobic organism implicated in intra-abdominal infections. However, 2 mg/L is often cited as target exposure in clinical practice (15) owing to a natural MIC distribution of *B. fragilis* that is almost always <2 mg/L (16). We also included the AUC_0–24,ss_/MIC targets of 2 and 4 µg/mL as reported in studies of adults and children evaluating target attainment of metronidazole for *B. fragilis* (16, 17). For each pharmacodynamic target, we calculated the proportion of SCAMP participants who met the target, stratified by age, defined as PMA in weeks or age group based on PMA (<34, 34–40, >40 weeks) and PNA (<14 and ≥14 days).

### Clinical efficacy outcomes

We leveraged the clinical efficacy outcomes used in the SCAMP trial, which included therapeutic success (defined as the absence of death, negative bacterial blood cultures within 30 days after the last dose of study drug, and presumptive clinical cure at 30 days), clinical cure (composite score including indicators of illness severity: fraction of inspired oxygen, urine output, cardiovascular inotrope support, need for mechanical ventilation, presence of seizure, and lowest serum pH) (18), and time to full enteral feeds (≥100 mL/kg/d).

### Safety outcomes

Pre-specified safety outcomes of interest in the SCAMP trial were dichotomous and included progression to a higher Bell’s stage of necrotizing enterocolitis (19), intestinal strictures, intestinal perforation, positive blood culture (bacterial or fungal), seizures, death, and intraventricular hemorrhage grade 3 or 4.

The exposure–response relationship between simulated metronidazole exposures (C_max,ss_, C_min,ss_, AUC_0–24,ss_, and AUC_cum_) and clinical efficacy and safety outcomes was evaluated for each SCAMP trial participant and their dosing regimen. The number and proportion of participants with efficacy and safety outcomes at three different low and high exposure levels (≤10th vs >90th percentile; ≤25th vs >75th percentile; and ≤50th vs >50th percentile) for each simulated exposure parameter were determined and compared using Fisher’s exact tests. Summary statistics for continuous outcome measures were calculated at low and high exposure levels and compared using a two-tailed Student’s *t*-test or Wilcoxon rank sum test, as appropriate. Logistic regression adjusted for GA was used to assess the relationship between exposures and outcomes. *P* values <0.05 were considered statistically significant. Simulations were performed using NONMEM version 7.4, and all other manipulation and visualization were performed using STATA (version 15.1, College Station, TX), R (version 3.4.1, R Foundation for Statistical Computing, Vienna, Austria), and RStudio (version 1).

## RESULTS

### Exposure simulations and surrogate pharmacodynamics

Simulated metronidazole exposures are shown in Table 1. At steady state, more than 99% of infants in SCAMP achieved the surrogate pharmacodynamics target C_min,ss_ > 8 mg/L, and 100% of infants had C_min,ss_ > 2 mg/L. No differences were observed when examined by PMA. After the loading dose, the percentage of SCAMP participants who had predicted metronidazole trough concentrations (C_min,1_) >8 and >2 mg/L were 35% and 100%, respectively. All participants exceeded the target AUC_0–24,ss_/MIC ratio ≥ 70 for MICs of 2 and 4 µg/mL at the dosages received in SCAMP (Table 1).

**TABLE 1:** Simulated metronidazole exposure for critically ill infants in the SCAMP trial^*a*^

	PNA group (days)		PMA group (weeks)			Total
	**<14**	**≥14**	**<34**	**34–40**	**>40**	
*N*	69	53	54	54	14	122
C_max,1_ (mg/L)	21.31 (5.17)	19.76 (4.04)	18.69 (4.75)	21.85 (3.77)	23.5 (5.45)	20.64 (4.75)
C_min,1_ (mg/L)	8.20 (2.97)	6.78 (1.89)	8.78 (2.62)	6.72 (2.07)	6.31 (2.95)	7.59 (2.64)
AUC_0–24,1_ (mg*h/L)	324.28 (78.06)	287.74 (52.13)	311.07 (75.87)	305.46 (59.50)	309.43 (88.64)	308.40 (70.17)
C_max,ss_ (mg/L)	35.32 (10.94)	31.69 (11.01)	31.41 (10.55)	33.58 (8.64)	43.38 (16.16)	33.74 (11.07)
C_min,ss_ (mg/L)	25.14 (9.74)	22.30 (10.48)	22.39 (9.86)	23.22 (7.58)	32.44 (15.26)	23.91 (10.12)
AUC_0–24,ss_ (mg*h/L)	718.52 (247.81)	640.85 (258.07)	638.90 (244.78)	674.06 (194.58)	903.14 (377.23)	684.78 (254.23)
AUC_0–24, ss_/MIC ≥ 70 for MIC = 2 µg/mL (%)	100	100	100	100	100	100
AUC_0–24, ss_/MIC ≥ 70 for MIC = 4 µg/mL (%)	100	100	100	100	100	100
C_min,1_ > 8 (%)	45	23	59	15	21	35
C_min,1_ > 2 (%)	100	100	100	100	100	100
C_min,ss_ > 8 (%)	99	100	98	100	100	99
C_min,ss_ > 2 (%)	100	100	100	100	100	100

^*a*^ AUC_0–24,1_, area under the concentration–time curve from 0 to 24 h after loading dose; AUC_0–24,ss_, steady state area under the concentration–time curve from 0 to 24 h; C_max,1_, maximum concentration after loading dose; C_max,ss_, maximum concentration at steady state; C_min,1_, minimum concentration after loading dose; C_min,ss_, minimum concentration at steady state; PMA, postmenstrual age; PNA, postnatal age. Data are mean (SD) unless otherwise specified.

### Clinical efficacy outcomes

Out of 122 infants in SCAMP, 101 (82%) had therapeutic success based on SCAMP trial specification (the absence of death, negative bacterial blood cultures within 30 days after the last dose of study drug, and presumptive clinical cure at 30 days). Exposures by clinical efficacy outcomes are reported in Table S1. In unadjusted analysis, SCAMP participants who achieved therapeutic success had higher AUC_0–24,ss_, C_max,ss_, C_min,ss_, and AUC_cum_ than infants who did not achieve therapeutic success (mean AUC_0–24,ss_: 690.1 vs 524.6 µg*h/mL, *P* = 0.01; mean C_max,ss_: 34.2 vs 26.5 µg/mL, *P* = 0.01; mean C_min,ss_: 23.9 vs 17.8 µg/mL, *P* = 0.02; mean AUC_cum_: 5137.3 vs 3211.6 µg*h/mL, *P* = 0.03). However, after logistic regression adjusted for GA, the differences in odds ratios (ORs) for therapeutic success were not statistically significant (Table 2). No difference was observed for clinical cure (Table 2), nor time to full enteral feeds (Fig. S1).

**TABLE 2:** Relationship between metronidazole exposure and clinical events of efficacy or safety^*a*^^,^*^b^*

Outcome/event	Steady state AUC_0–24,ss_ (µg*h/mL)		Steady state C_max_ (µg/mL)		Steady state C_min_ (µg/mL)	
	Odds ratio (95% CI)	*P*	Odds ratio (95% CI)	*P*	Odds ratio (95% CI)	*P*
Efficacy outcome						
Clinical cure	1.002 (0.999–1.006)	0.181	1.06 (0.975–1.159)	0.166	1.060 (0.970–1.159)	0.196
Therapeutic success	1.003 (0.999–1.007)	0.099	1.087 (0.989–1.195)	0.084	1.080 (0.981–1.189)	0.115
Safety outcome						
Any safety event	0.998 (0.996–1.000)	0.063	0.951 (0.901–1.000)	0.05	.0957 (0.911–1.005)	0.080
Progression to higherstage of necrotizing enterocolitis	0.994 (0.985–1.004)	0.258	0.887 (0.710–1.108)	0.289	0.864 (0.677–1.102)	0.238
Intestinal strictures	0.997 (0.991–1.004)	0.382	0.937 (0.805–1.100)	0.397	0.928 (0.789–1.092)	0.369
Intestinal perforation	1.000 (0.996–1.004)	0.956	0.997 (0.908–1.094)	0.942	0.997 (0.903–1.101)	0.954
Positive blood culture	0.999 (0.997–1.002)	0.466	0.976 (0.920–1.036)	0.424	0.979 (0.921–1.041)	0.493
Seizure	1.000 (0.997–1.004)	0.942	0.999 (0.921–1.036)	0.998	1.005 (0.923–1.095)	0.908
Death	0.997 (0.992–1.002)	0.212	0.924 (0.825–1.035)	0.171	0.937 (0.838–1.048)	0.253
IVH	0.998 (0.992–1.004)	0.460	0.946 (0.820–1.092)	0.449	0.948 (0.819–1.098)	0.476

^*a*^ AUC_24,ss_, steady state area under the concentration–time curve from 0 to 24 h; C_max_, maximum drug concentration; C_min_, minimum drug concentration; IVH, intraventricular hemorrhage.

^*b*^ Odds ratios and 95% confidence intervals were calculated using logistic regression with exposure as a continuous variable, adjusted for gestational age in weeks.

### Safety outcomes

A total of 31 (29%) SCAMP participants had at least one SCAMP trial pre-specified safety event. There were 2 participants with progression to a higher Bell’s stage of necrotizing enterocolitis, 3 participants with intestinal strictures, 4 participants with intestinal perforation, 14 participants with positive blood culture (bacterial or fungal), 5 participants with seizures, 5 participants with death, and 2 participants with intraventricular hemorrhage grade 3 or 4. Exposures by safety outcomes are reported in Table S1. SCAMP participants with at least one safety event had lower, but still relatively high, metronidazole exposures compared with participants without any safety events (mean AUC_0–24,ss_: 577.9 vs 695.2 µg*h/mL; mean C_max,ss_: 29.0 vs 34.6 µg/mL; mean C_min,ss_: 19.8 vs 24.1 µg/mL). ORs for all safety events were not significant in unadjusted analysis nor analysis adjusted for GA (Table 2).

## DISCUSSION

In the first combined exposure–response and safety analysis of metronidazole using prospective clinical trial data in preterm and term infants, metronidazole dosing achieved simulated exposure targets and exposure did not relate to clinical safety events. This study also represents the first application of our optimized population popPK model of metronidazole (12) in a relevant population. Study data were submitted to the United States Food and Drug Administration, which led to a labeling update and addition of a new indication to the metronidazole package insert for infants <4 months of age with intra-abdominal infections (20–23).

We found the weight- and PMA-based dosing regimen in SCAMP resulted in nearly 100% of participants achieving targets of AUC_0–24,SS_/MIC ≥ 70 at MIC 2 µg/mL and MIC 4 µg/mL, targets associated with efficacy in other studies of IV metronidazole, including pediatric abdominal infections (16, 17, 24). Infants with therapeutic success had, on average, higher metronidazole exposures compared with infants without therapeutic success; however, exposures did not predict therapeutic success in logistic regression analyses adjusting for GA. No significant relationships between metronidazole exposure or other clinical efficacy events were identified. The lack of statistically significant relationships may be explained by generally high exposures, well in excess of proposed clinical therapeutic targets (15). The relationship between metronidazole exposure and therapeutic success may have been confounded by age- or prematurity-related risks. In addition, efficacy outcomes could be influenced by other antibiotics or underlying pathology, as SCAMP included infants with a variety of suspected and confirmed infections and a range of disease severity (13).

We found no evidence of a relationship between metronidazole exposure and the occurrence of any safety event despite generally high exposures. This lack of relationship may be related to the overall favorable safety profile of metronidazole and low number of individual safety events. This exposure–safety analysis is limited to pre-specified safety events of interest and does not comprehensively evaluate all potential adverse events, but generally supports the safety of metronidazole at the dosing used in SCAMP (13, 25). Given the high simulated exposures and vulnerable population, additional studies should continue to evaluate the short- and long-term safety of metronidazole.

A major strength of this study is the inclusion of several surrogate pharmacodynamic endpoints, as prior studies use single and/or varied surrogate pharmacodynamic targets (2, 6, 10) or a single clinical safety event of interest (11). Another strength of this study is the inclusion of clinical efficacy and safety outcomes.

Our study has important limitations. Participant heterogeneity in age, disease severity, and concurrent treatments may have limited the ability to detect significant differences in uncommon safety outcomes. SCAMP originally enrolled critically ill preterm neonates with complicated intra-abdominal infections; however, due to enrollment challenges, term and older infants with a range of infections were later included in the trial. However, SCAMP is the largest prospective randomized clinical trial of infants with complicated intra-abdominal infections to date (13) and is sized comparably to trials in similar populations with complicated infections (26), supporting our findings. We simulated, rather than directly measuring, exposures, which was necessary because of the high variability in metronidazole PK (6) and the opportunistic sampling design in SCAMP. Although the popPK model likely predicts reliable exposures because the model was validated and optimized in this population and accounts for the influence of age and weight on PK parameters (12), our findings could be further strengthened with additional studies that directly observe exposures and corresponding outcomes. Another important limitation of this study is that SCAMP participants received multimodal antimicrobial therapy; therefore, all findings must be interpreted within this context.

Overall, our findings support the metronidazole IV dosing by PMA in term and preterm infants as previously recommended by Cohen-Wolkowiez et al. (10) and as follows: 15 mg/kg loading dose followed by maintenance doses of 7.5 mg/kg every 12 h for PMA < 34 weeks, every 8 h for PMA 34–40 weeks, and every 6 h for PMA > 40 weeks.

## Data Availability

To help expand the knowledge base for pediatric medicine, the PTN is pleased to share data from its completed and published studies with interested investigators. For requests, please contact a PTN Program Manager (PTN-Program-Manager@dm.duke.edu).

## SUPPLEMENTAL MATERIAL

Supplemental material aac.00377-25-s0001.pdf Table S1; Fig. S1.

ASM does not own the copyrights to Supplemental Material that may be linked to, or accessed through, an article. The authors have granted ASM a non-exclusive, world-wide license to publish the Supplemental Material files. Please contact the corresponding author directly for reuse.
