# Supplementary material for: Metronidazole exposure–response and safety in infants
Source: Antimicrob Agents Chemother. 2025 Sep 19;69(11):e00377-25. doi: 10.1128/aac.00377-25 (PMC12587542; doi:10.1128/aac.00377-25)
Supplement: Supplemental material — Table S1; Fig. S1. [file aac.00377-25-s0001.pdf]

Metronidazole Exposure-Response and Safety in Infants

Supplemental Materials

Rachel L. Randell MD, MSCR[a,b]; Stephen J. Balevic MD, PhD[a,b]; Rachel G. Greenberg MD, MB, MHS[a,b]; Michael Cohen-Wolkowiez MD, PhD[a,b]; Michael J. Smith MD, MSCE[a]; Daniel K. Benjamin, Jr. MD, PhD[a,b]; Catherine Bendel, MD[c]; Joseph M. Bliss, MD, PhD[d]; Hala Chaaban, MD[e]; Rakesh Chhabra, MD[f]; Christiane E.L. Dammann, MD[g]; L. Corbin Downey, MD, MPH[h]; Chi Hornik[a,b]; Naveed Hussain, MBBS, DCH[i]; Matthew M. Laughon, MD, MPH[j]; Adrian Lavery MD, MPH[k]; Fernando Moya, MD[l]; Matthew Saxonhouse, MD[m]; Gregory M. Sokol, MD[n]; Andrea Trembath MD, MPH[o]; Joern-Hendrik Weitkamp, MD[p]; Christoph P. Hornik MD, PhD, MPH[a,b]; on behalf of the Best Pharmaceuticals for Children Act – Pediatric Trials Network Steering Committee

[a]Department of Pediatrics, Duke University, Durham, NC, USA; [b]Duke Clinical Research Institute, Durham, NC, USA; [c]Department of Pediatrics, University of Minnesota Medical School, Minneapolis, MN, USA; [d]Division of Neonatology, Department of Pediatrics, University of Rochester Medical Center, Rochester, NY, USA; [e]Division of Neonatology, Dept. of Pediatrics, Oklahoma University Health Sciences Center, Oklahoma City, OK, USA; [f]Division of Neonatology, Department of Pediatrics, Hackensack University Medical Center, Hackensack Meridian School of Medicine, Hackensack, NJ, USA; [g]Department of Pediatrics, Tufts Medical Center, Tufts University, Boston, MA, USA; [h]Department of Pediatrics, Atrium Health Wake Forest Baptist Medical Center, Winston-Salem, NC, USA; [i]Division of Neonatology, Dept. Of Pediatrics, Connecticut Children's, Hartford, CT, USA; [j]Department of Pediatrics, Division of Neonatal-Perinatal Medicine, The University of North Carolina at Chapel Hill, Chapel Hill, NC, USA; [k]Children's Hospital of Orange County, Orange, CA, USA; [l]Pediatrix Medical Group, Austin, TX, USA; [m]Division of Neonatology, Department of Pediatrics, Levine Children's Hospital, Wake Forest School of Medicine, Charlotte campus, Atrium Healthcare, Charlotte, NC, USA; [n]Department of Pediatrics, Indiana University School of Medicine, Indianapolis, IN, USA; [o]Division of Neonatal-Perinatal Medicine, University of North Carolina at Chapel Hill, Chapel Hill, NC, USA; [p]Mildred Stahlman Division of Neonatology, Monroe Carell Jr. Children's Hospital at Vanderbilt, Vanderbilt University Medical Center, Nashville, TN, USA.

CONTENTS

Supplemental Table S1 2

Supplemental Figure S1 3

SUPPLEMENTAL TABLE S1 Summary statistics of metronidazole exposure by efficacy and safety endpoints

Outcome/Event	Steady state C_{max} (µg/mL)			Steady state C_{min} (µg/mL)			Steady state AUC $_{0\text{-}24,ss}$ (µg*h/mL)			Cumulative AUC (µg*h/mL)		
	N	Mean (SD)	Median (range)	N	Mean (SD)	Median (range)	N	Mean (SD)	Median (range)	N	Mean (SD)	Median (range)
**Efficacy outcome**												
Therapeutic success	101	34.21 (10.79)	32.27 (14.0-80.3)	101	23.94 (9.68)	21.89 (3.27-63.7)	101	690.11 (245.79)	644.79 (177.5-1722)	101	5137.30 (2359.6)	4680 (697-13000)
No therapeutic success	9	26.52 (11.41)	26 (9.14-51.9)	9	17.84 (10.64)	15.6 (3.05-42.6)	9	524.58 (266.12)	488 (133.5-1130)	9	3211.56 (2015.19)	3040 (244-5860)
**Safety outcome**												
Overall	122	33.14 (11.09)	31.90 (9.14-80.3)	122	22.97 (10.13)	21.43 (3.05-63.7)	122	665.37 (255.22)	627.21 (133.5-1722)	122	4747.85 (2437.35)	4455 (244-13000)
Any safety event	31	28.96 (8.28)	28.795 (9.14-45.5)	31	19.80 (7.50)	19.55 (3.05-35.92)	31	577.92 (189.83)	594.8 (133.5-974)	31	4036.26 (2068.95)	3900 (244-7760)
No safety event	91	34.56 (11.59)	32.26 (14.0-80.3)	91	24.05 (10.70)	21.52 (3.27-63.7)	91	695.16 (268.40)	633.67 (177.5-1722)	91	4990.26 (2515.20)	4540 (645-13000)
NECP	2	26.6 (0.85)	26.6 (26-27.2)	2	16 (0.57)	16 (15.6-16.4)	2	500.5 (17.68)	500.5 (488-513)	2	2002 (2486.19)	2002 (244-3760)
Intestinal strictures	3	27.90 (7.61)	28.58 (19.98-35.15)	3	17.84 (5.65)	17.48 (12.39-23.66)	3	540.23 (157.66)	542.47 (381.5-696.8)	3	4870 (1978.86)	4600 (3040-6970)
Intestinal perforation	4	32.04 (5.03)	31.97 (26.83-37.39)	4	22.30 (4.39)	22.46 (16.91-27.39)	4	645.50 (111.95)	646.55 (516.89-772)	4	6057.5 (1115.45)	6330 (4600-6970)
Positive blood culture	14	30.45 (9.830596)	30.93 (15.41-45.5)	14	20.95 (8.68)	20.52 (7.47-35.92)	14	609.68 (222.35)	609.5 (264.63-974)	14	3900.71 (2201.85)	3795 (910-7760)
Seizure	5	32.02 (8.047312)	31.08 (22.7-44.85)	5	23.02556 (6.760586)	22.00909 (15.0-33.75)	5	654.74 (176.68)	624.42 (447-937)	5	4092 (2045.08)	4610 (910-6160)
Death	5	25.53 (9.60)	27.70 (9.14-32.54)	5	17.32 (8.81)	19.16 (3.05-24.89)	5	506.92 (224.02)	558 (133.5-684.86)	5	2264.8 (2092.11)	1570 (244-5570)
Survivors	117	33.47 (11.07)	31.91 (14.0-80.3)	117	23.21 (10.14)	21.44 (3.27-63.7)	117	672.14 (255.13)	630 (177.5-1722)	117	4853.97 (2401.81)	4540 (645-13000)
IVH	2	27.88 (9.56)	27.88 (21.12-34.64)	2	19.26 (12.46)	19.26 (10.45-28.06)	2	557.11 (272.35)	557.11 (364.53-749.68)	2	5085 (2411.234)	5085 (3380-6790)

Abbreviations: AUC, area under the concentration-time curve; $AUC_{24,ss}$, steady state area under the concentration-time curve from 0 to 24 hours; C_{max}, maximum drug concentration; C_{min}, minimum drug concentration; IVH intraventricular hemorrhage; NECP, progression to higher stage of necrotizing enterocolitis.

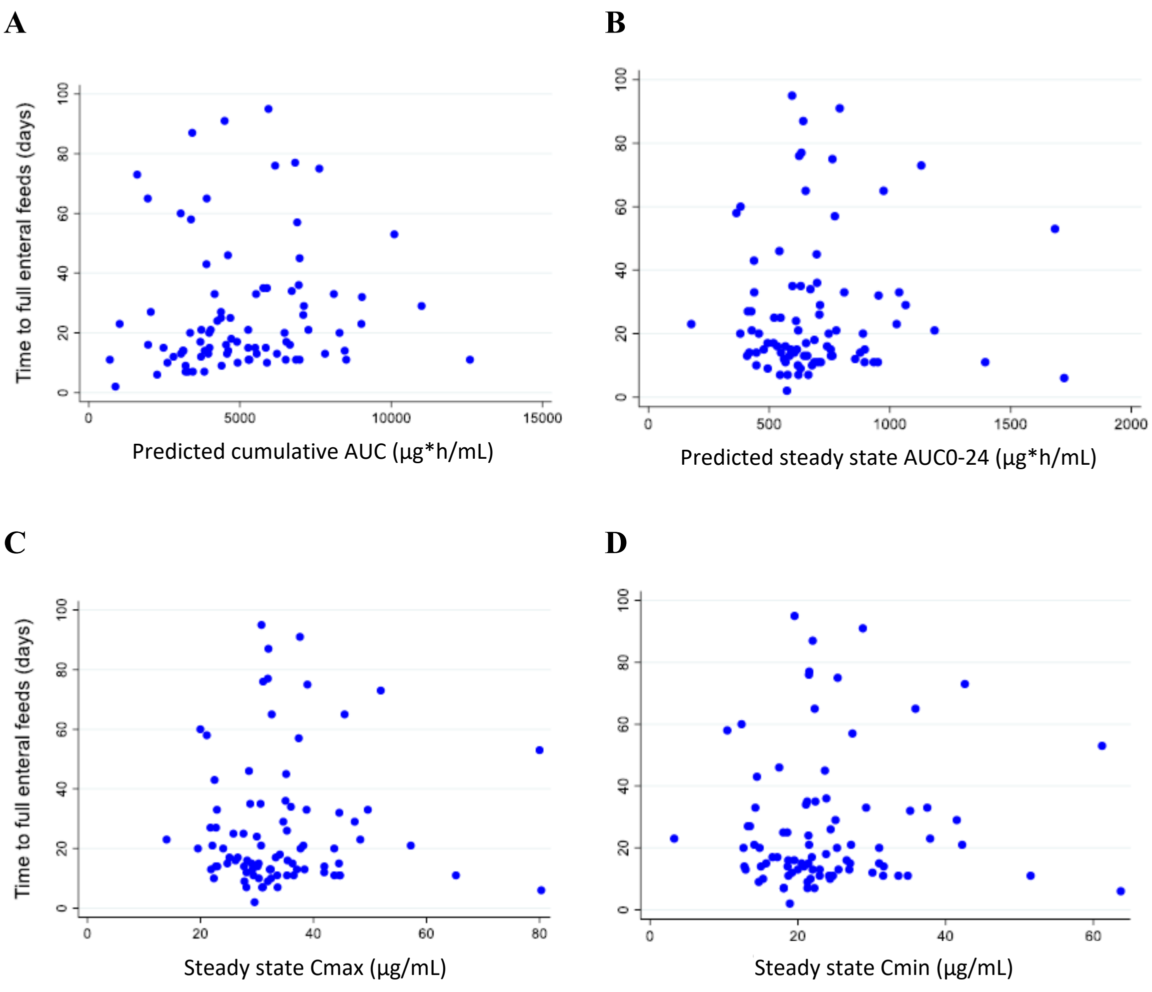

SUPPLEMENTAL FIGURE S1 Plots of simulated metronidazole exposure and time to full enteral feeds, with exposure represented as (A) AUC_{cum}, (B) $AUC_{0\text{-}24,ss}$, (C) $C_{max,ss}$, and (D) $C_{min,ss}$.
